# The Head AIS 4+ Injury Thresholds for the Elderly Vulnerable Road User Based on Detailed Accident Reconstructions

**DOI:** 10.3389/fbioe.2021.682015

**Published:** 2021-06-23

**Authors:** He Wu, Yong Han, Di Pan, Bingyu Wang, Hongwu Huang, Koji Mizuno, Robert Thomson

**Affiliations:** ^1^School of Aeronautics and Astronautics, Xiamen University, Xiamen, China; ^2^School of Mechanical and Automotive Engineering, Xiamen University of Technology, Xiamen, China; ^3^Department of Mechanical Science and Engineering, Graduate School of Engineering, Nagoya University, Nagoya, Japan; ^4^Chalmers University of Technology, Gothenburg, Sweden

**Keywords:** the elderly, accident reconstruction, video information, head injury criteria, vulnerable road user

## Abstract

Compared with the young, the elderly (age greater than or equal to 60 years old) vulnerable road users (VRUs) face a greater risk of injury or death in a traffic accident. A contributing vulnerability is the aging processes that affect their brain structure. The purpose of this study was to investigate the injury mechanisms and establish head AIS 4+ injury tolerances for the elderly VRUs based on various head injury criteria. A total of 30 elderly VRUs accidents with detailed injury records and video information were selected and the VRUs’ kinematics and head injuries were reconstructed by combining a multi-body system model (PC-Crash and MADYMO) and the THUMS (Ver. 4.0.2) FE models. Four head kinematic-based injury predictors (linear acceleration, angular velocity, angular acceleration, and head injury criteria) and three brain tissue injury criteria (coup pressure, maximum principal strain, and cumulative strain damage measure) were studied. The correlation between injury predictors and injury risk was developed using logistical regression models for each criterion. The results show that the calculated thresholds for head injury for the kinematic criteria were lower than those reported in previous literature studies. For the brain tissue level criteria, the thresholds calculated in this study were generally similar to those of previous studies except for the coup pressure. The models had higher (>0.8) area under curve values for receiver operator characteristics, indicating good predictive power. This study could provide additional support for understanding brain injury thresholds in elderly people.

## Introduction

The Global Status Report on Road Safety (2018) shows that 1.35 million people die each year from road traffic accidents (World Health Organization, 2018) and that more than half of the global deaths were vulnerable road users (VRUs) (specifically 23% of pedestrians, 3% of cyclists, and 28% of motorized 2–3 wheelers). In China, there were 63,772 deaths caused by traffic accidents in 2017, in which elderly people (the age ≥ 60 years) accounted for 30.35% (TABC, 2017).

Brain injuries have been observed as the most fatal factor to the VRUs and have been investigated thoroughly in the past five decades (Gadd, 1966; Nahum et al., 1977; Ward et al., 1980; Hertz, 1993; Arbogast et al., 1995; Hardy et al., 2001; Melvin and Lighthall, 2002; Shi et al., 2020). Due to the complexity of the head anatomical structure, many head injury tolerances (Nusholtz et al., 1984; Margulies and Thibault, 1992; Bain and Meaney, 2000; Zhang et al., 2004) and head injury criteria (HIC) (Versace, 1971; Newman, 1986; Newman and Shewchenko, 2000; Willinger and Baumgartner, 2003; Marjoux et al., 2008; Takhounts et al., 2011, 2013; Kimpara and Iwamoto, 2012) have been proposed for evaluating the human head injury risk under various crash conditions. Two types of HIC have been proposed for evaluating head injury risk; one is based on head kinematics and the other on local tissue stress and strain information. Kinematic-based criteria include the head injury criterion (HIC) (National Highway Traffic Safety Administration (NHTSA), 1972), the Brain Injury Criteria (BRIC) (Takhounts et al., 2011, 2013), the Generalized Acceleration Model for Brain Injury Threshold (GAMBIT) (Newman, 1986), and the head impact power (HIP) (Newman and Shewchenko, 2000). The development of computer technology and finite element (FE) head models facilitated brain tissue-based injury criteria such as the von Mises stress, shear stress (Donnelly and Medige, 1997; Kang et al., 1997; Darvish and Crandall, 2001), pressure, the maximal principal strain (MPS), the cumulative strain damage measure (CSDM) (Bandak and Eppinger, 1994; Takhounts et al., 2003), and the dilatation damage measure (DDM) (Nusholtz et al., 1995). For the elderly, as the brain size decreases and the subdural space increases (Genarelli and Thibault, 1982), the relative motion between the skull and the brain increases significantly under various impact conditions, which would lead to a greater risk of vein rupture and hematoma (Kleiven and Holst, 2001; Richards and Carroll, 2012). However, there are few studies on the head injury tolerances for the elderly.

Brain injury criteria and mechanism tolerances based on biomechanical experiments (Melvin and Lighthall, 2002) and indepth accident reconstructions (Yao et al., 2008; Peng et al., 2012; Bourdet et al., 2014; Giordano and Kleiven, 2014; Nie and Yang, 2014; Sahoo et al., 2016) have been intensively investigated. Shi et al. (2020) investigated the effectiveness of the various HIC in the prediction of VRUs severe head injuries caused by ground impact in 10 accidents and showed that predictors like angular acceleration, linear acceleration, HIC, coup pressure, MPS, and CSDM had good capability to predict severe head injuries. However, the correlation between those injury predictors and injury risk still needs more analyzing. With more real-world accident cases collected and reconstructed with high accuracy, the purpose of the current study was to establish the head AIS 4+ injury tolerance of elderly people based on various HIC. A total of 30 detailed real-world elderly VRU accidents with video information from the TRaffic Accident database with Video (VRU-TRAVi) (Han et al., 2018; Shi et al., 2020) was used.

## Materials and Methods

### Vulnerable Road Users Accident Data

A total of 30 real-world VRU accident cases were selected and reconstructed from the VRU-TRAV database. This database was established in 2015, and more than 1,500 cases of video information have been collected at present. Among them, about 1,300 cases (only video information) were downloaded from the Internet (Youku, YouTube, Tencent, etc.). In addition, more than 220 in-depth accidents (contains video and detailed medical records) were obtained from National Automobile Accident In-Depth Investigation System (NAIS) and Academy of Forensic Science (AFS). NAIS and AFS meet the ethical procedures for incident data collection. We have intensive cooperation with NAIS workstations (Shanghai University of Engineering and Technology and Xihua University) and AFS to obtain these accident data. The selection standards for VRU accidents were:

(a) All cases were for the elderly (age ≥ 60 years).
(b) Each case has detailed accident sketches, vehicle damage photos, video information from the vehicle recorder or road monitoring, and detailed head injury reports.
(c) The contact area between the VRU’s head and the vehicle front-end structure (such as the A-pillar, bonnet, windshield, or ground) could be obtained from the above information.
(d) From the video records, the kinematic motion of the vehicle and the VRU kinematics before/during/after collisions could be observed clearly.
(e) The injury report should record details of the type of head injury and the severity of the head injuries having been classified and coded by using the maximum degree of injury severity (MAIS) (Association for the Advancement Automotive Medicine, 2005).

Table 1 shows the basic information of the 30 accidents (detailed information listed in Supplementary Table 1), in which the VRU’s age was mainly distributed between 60 and 80 years old, and the five most common types of head injuries (detailed head injury information listed in Supplementary Table 2) were subarachnoid hemorrhage (SAH), subdural hematoma (SDH), skull fracture (SF), soft tissue hematoma (STH), and scalp laceration (SL).

**TABLE 1:** Basic information of 30 accidents.

	VRU Types					Gender		Age		
	Pedestrian		Cyclist		ETWs*	Male	Female	61–70	71–80	>80
No. of cases	11		4		15	20	10	14	15	1
percentage	37%		13%		50%	67%	33%	47%	50%	3%

	**The type of head injury**					**Injury severity**		**MAIS for head**		
	**SAH**	**SDH**	**SF**	**STH**	**SL**	**Death**	**No-death**	**0–1**	**2–3**	**≥4**

No. of cases	13	13	13	8	7	21	9	4	6	20
percentage	24%	24%	24%	15%	13%	70%	30%	13%	20%	67%

*ETWs, electric two-wheelers.

### Accident Reconstruction

**FIGURE 1:**
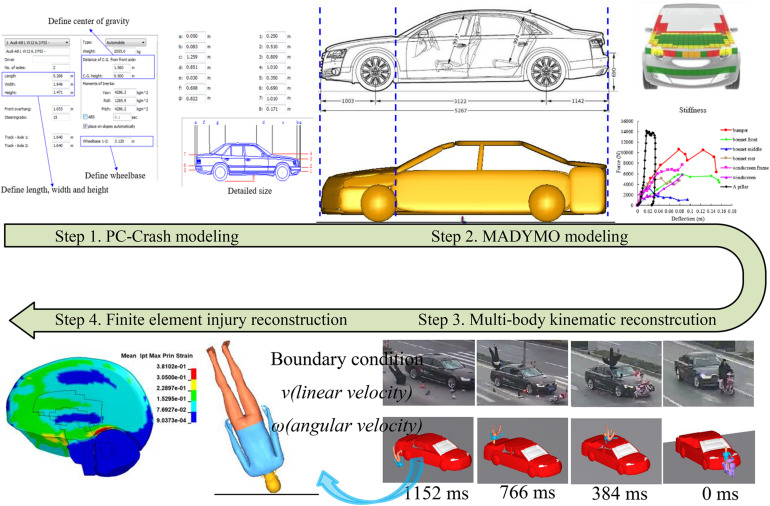
The methodology was implemented for the accident reconstruction.

Shi et al. (2020) described the accident reconstruction flow shown in Figure 1. There are four steps to reconstruct the kinematic and head injury severity of the VRUs by coupling multi-body system and FE models.

#### Step 1: PC-Crash Modeling

The multi-body vehicle and VRUs models were reconstructed based on vehicle and VRU size information in the accident files, and the estimation of vehicle speed was obtained by the video frame-by-frame analysis method (Han et al., 2019) and direct linear transformation (DLT) method (Han X. Y. et al., 2012). The initial impact position between vehicle and VRUs were mainly determined by the video and pictures of vehicle damage parts.

#### Step 2: MADYMO Modeling

The vehicle multi-body model used was developed based on the detailed vehicle structural dimensions using ellipsoids, and the front-end stiffnesses were defined based on Euro-NCAP test data (Martinez et al., 2007). For the pedal bicycle and electric two-wheelers, six hinges were used to simulate the motion between each component, and the stiffness characteristics were defined based on the studies of McLundie (2007) and Maki and Kajzer (2000). The VRU’s gender, stature, and weight were similarly reconstructed to the accident victims by using the scaling method on the baseline model of the 50th Chalmers Pedestrian Model (CPM) (Young, 1997; Yang et al., 2000). For the contact simulation, the elastic contact model was used to represent the contact between different multi-body models, and the friction coefficient was specified to be 0.2 between the VRU and the vehicles models, and 0.58 between the VRUs and the ground (Wood and Simms, 2000; Shi et al., 2018).

#### Step 3: Multi-body Kinematic Reconstruction

The final position of the vehicle and VRU was reconstructed based on the accident sketch by using PC-Crash and MADYMO code. The VRUs’ kinematic in both vehicle and ground contact were reproduced by comparing with the accident video information.

#### Step 4: Finite Element Injury Reconstruction

The head and torso boundary conditions pre-impact were defined by the output from running the multi-body kinematic reconstruction. These boundary conditions included three-axis linear and angular velocities of the head, chest, and pelvis centers of gravity (CG) and the relative position between the pedestrian to vehicle and ground impact. Some cases have both head-to-vehicle and head-to-ground impacts, some have only ground impacts, and the types of vehicles involved in the 30 cases are mainly sedan, SUV, and MPV. To make the FE vehicle model used for simulation match the dimensions of the accident vehicle as much as possible, a total of five FE vehicle models (Han X. Y. et al., 2012; Han Y. et al., 2012; Shi et al., 2018, 2019) were selected and used for the head-to-vehicle impacts simulations, and the ground surface was the asphalt road and defined as a rigid body (Tamura et al., 2014; Huang et al., 2020).

### Head Injury Criteria

All FE simulations were performed using the LS-DYNA MPP R9.3.0 (LSTC, Livermore, CA, United States) software. Eight HIC were computed with the THUMS V4.0.2 pedestrian model. The head kinematic-based criteria were the angular velocity, the angular and linear acceleration, and HIC (Versace, 1971; National Highway Traffic Safety Administration (NHTSA), 1972). The brain tissue level-based injury criteria were the coup pressure, MPS (Thibault et al., 1990; Bain and Meaney, 2000), and CSDM (Bandak and Eppinger, 1994; Takhounts et al., 2003). The estimated injury risks were compared with the injury records with AIS codes, and their effectiveness to predict severe head injuries was examined.

### Statistical Analysis

In this study, a single logistic regression method was used to establish the relationship between the head AIS 4+ injury risk and different evaluation criteria in the elderly. The injury risk curves are a sigmoid function derived based on Eq. 1 as follows:

(1) $$P\left(x\right)=\frac{1}{1+e^{-\left(\alpha_{0}+\alpha_{1}x\right)}}$$

Where *P*(*x*) is the probability of head AIS 4+ injuries for a value of injury criterium lower than or equal to *x*, α_*0*_ is the intercept, and α_*1*_ is the regression coefficient of *x*. Receiver operating characteristic (ROC) curves and area under curves (AUC) were further used to assess the predictive capability of the regression models. In this study, we used a confusion matrix to obtain the ROC curves and AUC. Confusion matrix (Li, 2012) is a concept from machine learning and is a measure of the performance of a classification model, which has two dimensions, one of which represents the actual value and the other the predicted value. Table 2 shows the expression of the confusion matrix for a typical binary classification problem. True positive (TP) means that the actual value is positive and the predicted value is also positive. False negative (FN) means that the actual value is positive and the predicted value is negative. Similarly, False positive (FP) and True negative (TN) indicated that the actual values are negative, and the predicted values are positive and negative, respectively.

**TABLE 2:** The expression of the confusion matrix for a typical binary classification problem.

Confusion matrix		Predicted value	
		Positive	Negative
Actual value	Positive	True positive (TP)	False negative (FN)
	Negative	False positive (FP)	True negative (TN)

To plot the ROC curves, we first need to define two measures, namely false positive rate (FPR) and true positive rate (TPR). FPR refers to the ratio of false-positive cases (the cases that predicted positive but are actually negative) out of all negative cases, it is defined by:

(2) $$\mathrm{FPR}=\frac{\text{FP}}{\text{FP}+\text{TN}}$$

True positive rate refers to the ratio of true-positive cases (the cases that predicted positive and actually are positive too) out of all positive cases, it is defined by:

(3) $$\text{TPR}=\frac{\text{TP}}{\text{TP}+\text{FN}}$$

In the binary classification task, the classification result can be obtained by setting a threshold. If the predicted value is higher than the threshold, it is classified as positive, and classified as negative if lower than the threshold. By setting different thresholds, we can get different confusion matrices, and then multiple pairs of FPR and TPR values can be calculated with FPR as the X-axis and TPR as the Y-axis, thus the ROC curve can be obtained by connecting them. The ROC indicates the predictive power with AUC 1.0 indicating a perfect model.

## Results

### Kinematic Response of Accident Reconstructions

**FIGURE 2:**
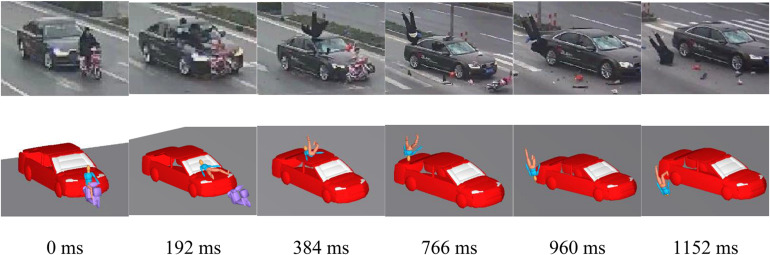
Comparison between the elderly reconstruction kinematics and the video records in case 9.

Based on the clear and complete accident video information, the kinematic response before/during/after the collision was reconstructed for a total of 30 elderly cases. Figure 2 shows the results of comparing the reconstruction kinematic response with the video information in case 9 (others are summarized in Supplementary Figure 1). The reconstructed pedestrian kinematics showed consistent results with the video records, including the relative position between the pedestrian and the vehicle, the pedestrian rotation angle (Shi et al., 2018), the pedestrian body region contact to the ground, the subsequent order of contacts (Han et al., 2018), and the final position (Wu et al., 2020). The reconstructed kinematic of the VRUs show consistency with the observed kinematics in the video records for all cases.

### Results of VRU Head Injury Simulations

**FIGURE 3:**
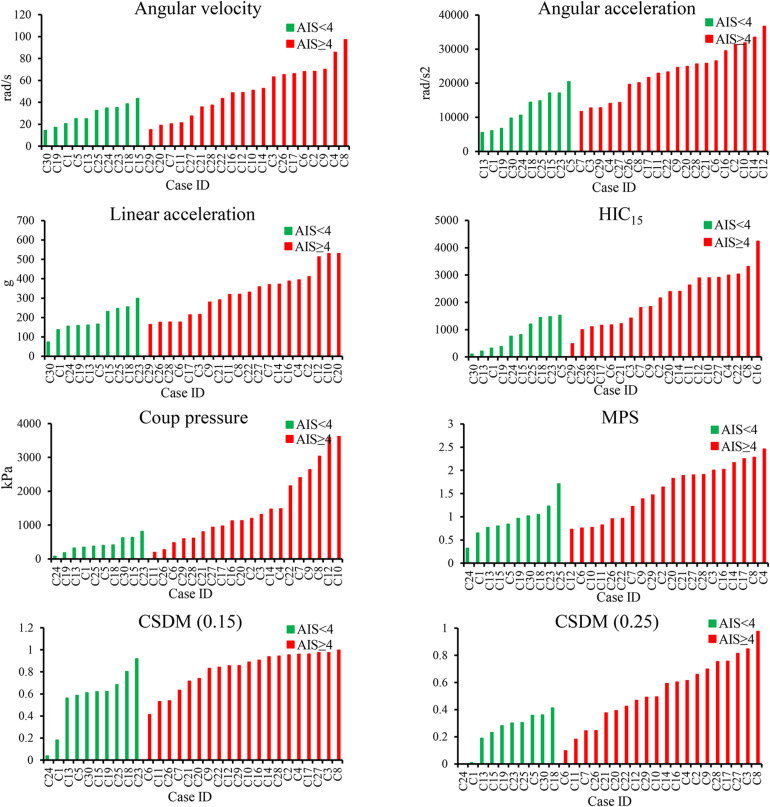
Simulated results of all head injury criteria.

For the 30 real-world VRU accident reconstructions, the simulated results of the four kinematic-based HIC and four brain tissue-based criteria are shown in Figure 3. The histograms were reordered in terms of the magnitude of the calculated injury criterion values according to the AIS < 4 cases (in the green columns) and the cases resulting in head AIS 4+ injuries (in the red columns). For each head kinematics-based and brain tissue-based criteria, the simulated values in green columns were globally lower than those simulated for the red columns. The ranges for the kinematic-based criteria consisting of the head angular velocity and acceleration, linear acceleration, and HIC_15_ were 14.4–97.3 rad/s, 5,550–36,688 rad/s^2^, 73–530.3 g, 103–4,238, respectively. The ranges for the brain tissue-based criteria consisting of the coup pressure, MPS, CSDM (0.15), and CSDM (0.25) were 78.44–3,618 kPa, 0.32–2.46, 0.04–0.996, and 0.001–0.98, respectively. The detailed parameter values for all head kinematics-based criteria and brain tissue-based criteria as determined from the simulations are listed in Supplementary Table 2.

### Injury Risk Curves for All Head Injury Criteria

**FIGURE 4:**
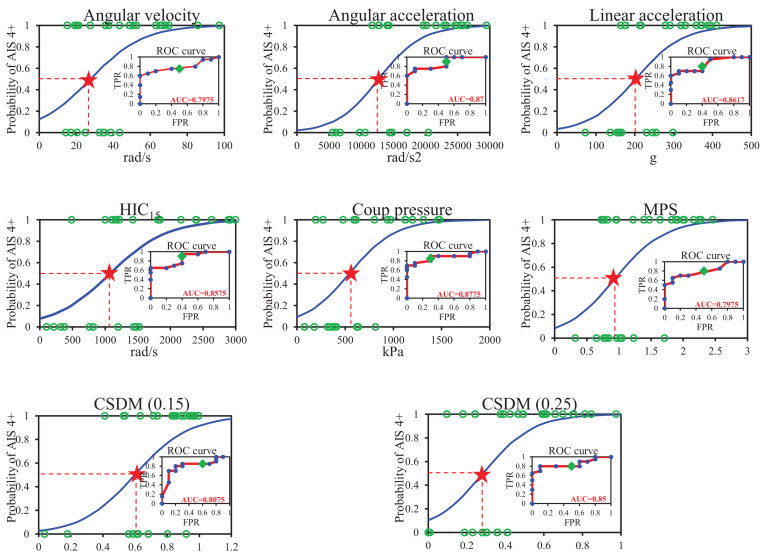
Head AIS 4+ injury risk curves for the head kinematic based criteria.

The injury risk curves for the four head kinematic based criteria and the four brain tissue based criteria were developed based on the regression of the histograms, and the resulting curves are shown in Figure 4, where the green circles are the experimental data, and the red pentagrams are the threshold at 50% AIS 4+ injury risk for each criterion. The subplots are the ROC curves, the blue dots are the FPR and TPR coordinates at different thresholds, and the green dots represent the FPR and TPR coordinates when the threshold is 0.5. The closer the ROC curve is to the upper left corner, and the closer the AUC = 1, the better the predictive capability of the regression equation. The AUC value for the kinematic-based criteria consisting of the head angular velocity and acceleration, linear acceleration, and HIC15 were 0.7975, 0.87, 0.8617, and 0.8575, respectively. Similarly, the AUC value for the brain tissue-based criteria consisting of the coup pressure, MPS, CSDM (0.15), and CSDM (0.25) were 0.8775, 0.7975, 0.8075, and 0.85, respectively. The logistic regression risk equations, the AUC value, and the 50% probability of head AIS 4+ injury for all HIC are summarized in Table 3.

**TABLE 3:** Summary of the results of head AIS 4+ injury risk curves.

Injury criteria	Risk curve equations for AIS 4+ injuries	AUC value	50% risk of AIS 4+	Reference value	Experimental materials
Angular vel	*P*(*x*) = 1/(1 + *e*^(−(−1.9372 + 0.0697*x*))^)	0.7975	27.8 rad/s	46.5 rad/s (Margulies and Thibault, 1992)	Animal studies, physical model and analytical model simulations
Angular acc	*P*(*x*) = 1/(1 + *e*^(−(−3.826 + 0.0003*x*))^)	0.87	12753 rad/s^2^	19000 rad/s^2^ (Chinn et al., 2001)	Accident reconstruction using Bimass head model
Linear acc	*P*(*x*) = 1/(1 + *e*^(−(−3.3202 + 0.0164*x*))^)	0.8617	202.5 g	250 g (Normalisation CED, 2011)	ATDs test
HIC_15_	*P*(*x*) = 1/(1 + *e*^(−(−2.4875 + 0.0023*x*))^)	0.8575	1,082	1,440 (National Highway Traffic Safety Administration (NHTSA), 1995)	Real-world accident cases
Coup pressure	*P*(*x*) = 1/(1 + *e*^(−(−2.3011 + 0.0042*x*))^)	0.8775	548 kPa	234 kPa (Ward et al., 1980)	Animal and human cadaver tests
MPS	*P*(*x*) = 1/(1 + *e*^(−(−2.4121 + 2.5618*x*))^)	0.7975	0.942	0.89 (Takhounts et al., 2013)	Animal tests
CSDM (0.15)	*P*(*x*) = 1/(1 + *e*^(−(−3.5831 + 5.9842*x*))^)	0.8075	0.6	0.55 (Takhounts et al., 2003)	Animal tests
CSDM (0.25)	*P*(*x*) = 1/(1 + *e*^(−(−2.1784 + 7.6546*x*))^)	0.85	0.285	0.25 (Takhounts et al., 2013)	Animal tests

## Discussion

### The Reliability of the Accident Reconstructions

The “accident reconstructions” using the real-world accident data to reproduce the collision process and human injuries can be used to alleviate the lack of real data to some extent (Kleiven, 2007; Yao et al., 2008). The traditional accident reconstruction methods were mostly based on police investigation records, including the objective vehicle trajectory traces developed from the investigation of the collision and the subjective information such as the comments and opinions garnered from the participants involved in the accident (Yao et al., 2008; Badea-Romero and Lenard, 2013). However, due to the lack of video information, factors exist regarding the uncertainty which influences the quality and reliability of the reconstruction. These include such factors as the VRUs’ kinematics, vehicle dynamics, impact area, impact angle, and landing posture, and the factors affecting the uncertainty could be alleviated by analyzing the videos for use in undertaking the accident reconstruction.

In the current study, the real-world VRU accidents with video information were selected and reconstructed by using a multi-body system (PC-Crash and MADYMO) and FE methods. Initially, the collision speed could be calculated more accurately using the video images and the DLT method. Then, the reconstructed kinematics could be verified against video frame by frame. Finally, the head impact conditions and injury outcomes could be more objectively compared with the hospital injury reports. In some cases (e.g., in case 18), it is difficult to observe the whole process of VRUs’ kinematic response after collision due to the perspective of the video; therefore, the kinematic response of the obscured part could be inferred by comparing the final position (Pascoletti et al., 2019a) and the observed kinematic response at the next moment. The 5th and 50th percentile THUMS models have different size and material properties, which could change the impact locations with the vehicle and injury severity of the head. But in this study, only the 50th percentile of THUMS was used for injury reconstruction in both male and female cases. The reasons are as follows: firstly, we used the CPM model to reconstruct the VRU’s kinematic response (including the impact location of the head), and the CPM model was scaled strictly according to the VRU’s height, weight, and gender in the real accident, and the reconstruction results were compared with the video information and vehicle damage photos. Then, the multi-body reconstruction results were input into the THUMS model as boundary conditions for injury reconstruction (shown in section “Accident Reconstruction”). Therefore, it can be ensured that the head-to-vehicle impact locations are consistent with the actual accident. Also, with the same loading boundary conditions (including the same linear and angular velocity, impact angle, and location), the little differences in the severity of head injury caused by the fifth and 50th THUMS models were observed, especially to simulate head impact with the ground.

### Regression Models Evaluation

**FIGURE 5:**
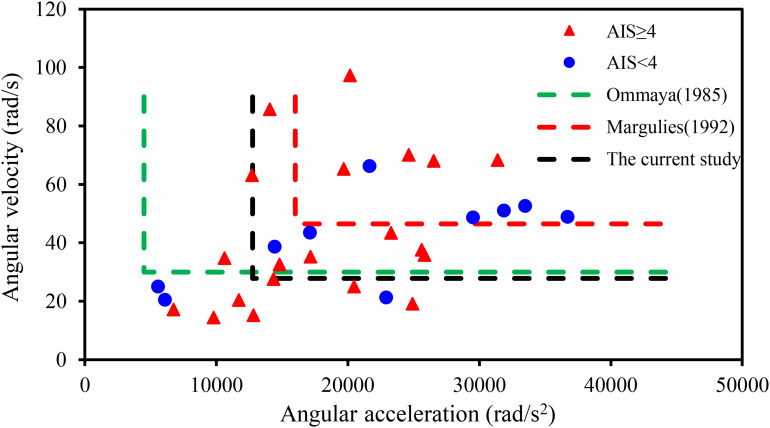
The relationship between head angular velocity, angular acceleration, and brain strain.

For the unbalanced sample of head injury level (the number of head AIS 4+ was 20 cases and no head AIS 4+ was 10 cases), the performance of the regression models was evaluated using ROC curves (shown in Figure 5) and AUC values (listed in Table 3) in this study. For all regression models, the values of AUC ranged from 0.8 to 0.88, indicating a good predictive capability. However, by comparing with previous studies (National Highway Traffic Safety Administration (NHTSA), 1995; Mertz et al., 1996), the initial probability (the probability when the horizontal coordinate is zero) of the regression model obtained in this study was slightly higher (the corresponding AIS 4+ probability was not zero (from 0.02 to 0.12) when the injury value was zero), and this phenomenon was one of the possible reasons why the AUC value could not be very close to 1. There are two reasons to explain this phenomenon: one is the insufficient sample size used to fit the regression model, and another is the unbalanced sample size and the number of on-head AIS 4+ only 10 cases. The main purpose of this study was to obtain the threshold of head AIS 4+ injury in elderly people, so the effect of the initial probability on the threshold was not significant, and the authors will subsequently increase the sample size further to obtain a more optimal regression model.

For the study of brain injuries tolerance, most human tolerance limits were constrained in the mild or moderate brain injuries (Rowson et al., 2012) because the head injury data used were mostly for football players, and there were limited data available with severe injuries, especially for the elderly. In this study, each criterion injury threshold of a 50% risk of an AIS 4+ severe brain injury for elderly people (listed in Table 3) was compared with those published in the literature for this field.

### Rotational Motion-Based Criteria (Angular Velocity and Angular Acceleration)

Based on animal experiments, Unterharnscheidt (1971) indicated that a rotational acceleration of 101–150 krad/s^2^ leads to no injury and when the accelerations up to 197 krad/s^2^, subdural hematomas combined with neurological injuries, could be observed. Ommaya (1985) used a primate model and suggested an injury threshold for sagittal plane rotation of the head of 4,500 rad/s^2^ when rotational velocity is less than 30 rad/s. Pincemaille et al. (1989), based on experimental data from volunteer boxers, found that the concussion thresholds for angular acceleration and angular velocity were in the range of 13.6–16, 25–48 rad/s, respectively. Margulies and Thibault (1992) utilized a primate model and proposed a DAI-tolerance limit (AIS 4+) for humans of 46.5 rad/s with an angular acceleration of 16,000 rad/s^2^. Patton et al. (2012) reported maximum rotational acceleration, respectively, a velocity of 4.5 krad/s^2^, 33 rad/s as a threshold for short or no loss of consciousness, based on a set of American football players’ head impact analyses. In this current study, the thresholds of angular velocity and angular acceleration (listed in Table 3) for the head injury level of AIS 4+ in the elderly were obtained based on logistic regression of the reconstruction results of 30 accidents, which were 27.8 rad/s and 12,753 rad/s^2^, respectively (shown in Figure 5). These thresholds were only similar to the concussion thresholds derived by Pincemaille et al. (1989) and Patton et al. (2012) and were much lower than those derived by Unterharnscheidt (1971) and Ono et al. (1980) for subdural hematoma and brain contusion. Those suggested that the probability of brain injury was higher in the elderly under the same impact conditions.

### Linear Motion-Based Criteria (Maximum Resultant Linear Acceleration and HIC)

**FIGURE 6:**
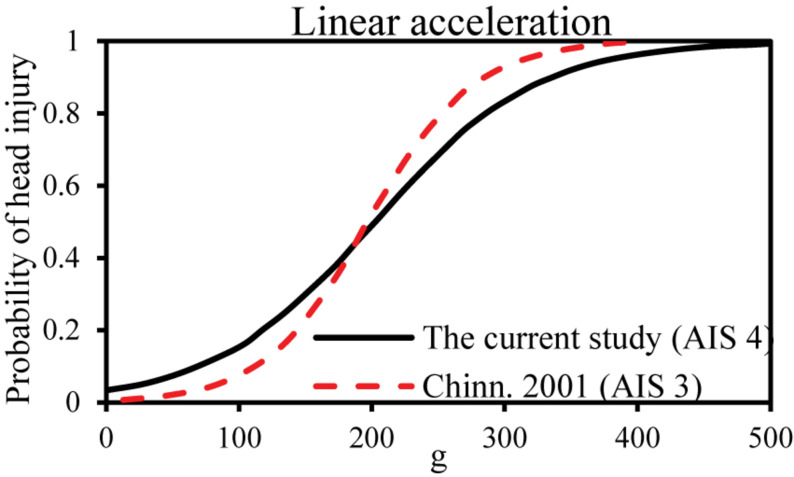
Comparison of the linear acceleration risk curves for head injury.

**FIGURE 7:**
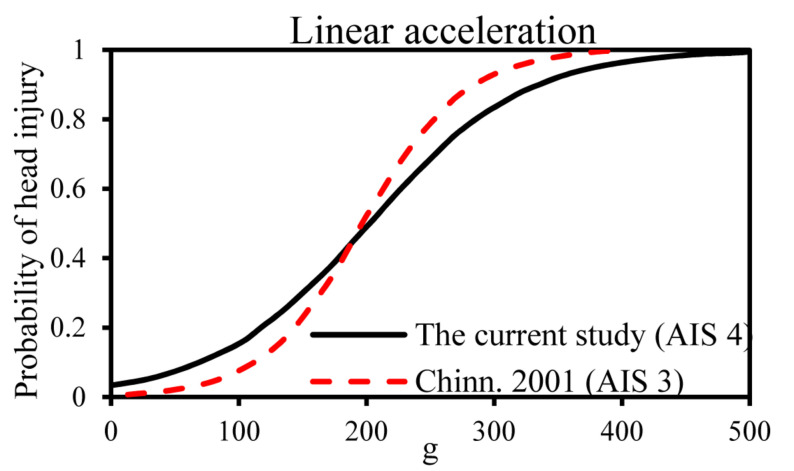
Comparison of the head injury criteria (HIC) risk curves for head AIS 4+ injury.

Early HIC were maximum resultant head acceleration because of their simplicity. The head accelerations of 200 and 250 g causing an AIS 3 and AIS 4 head injury were confirmed with previous studies (Newman, 1980; Chinn et al., 2001). However, this criterion does not take into account the time duration of the impact, so HIC was developed as a new HIC based on the Wayne state tolerance curve. The National Highway Traffic Safety Administration (National Highway Traffic Safety Administration (NHTSA), 1995) developed the HIC curves for various AIS injury levels, and HIC = 1,440 means a 50% probability of head AIS 4+ injury. Mertz et al. (1996) established a risk curve for HIC_15_ and skull fracture based on cadaver’s data and knowing that HIC = 1,420 means a 50% probability of skull fracture. The comparison of the linear acceleration and HIC risk curves for a head injury derived from these studies is shown in Figures 6, 7. In this current study, the critical value of 50% probability of head AIS 4+ injury for linear acceleration and HIC15 were 202.5 g and 1,082, respectively, which were slightly lower than (linear acceleration and HIC15 in this study were 19 and 24.86% lower than the earlier studies, respectively) the threshold of previous studies (Chinn et al., 2001; National Highway Traffic Safety Administration (NHTSA), 1995; Mertz et al., 1996).

### Stress-Based Criteria

Ward et al. (1980) simulated the head impacts in animal and human cadaver tests in aircraft accidents by using an original FE brain model and showed that the serious and fatal injuries would occur when the intracranial pressure exceeded 234 kPa. In the current study, the critical value of the pressure for a 50% risk brain injury was 548 kPa, which is much higher (the pressure threshold in this study was 134.2% higher) than Ward’s and his colleagues’ study. One reason for this large difference may be the difference in the material properties used, and another may the intense head-to-ground impact due to the ground being considered as a rigid body.

*For the strain-based criteria*, such as MPS and CSDM, Takhounts et al. (2003, 2011, 2013) proposed the 50% thresholds for MPS, CSDM0.15, and CSDM0.25 to predict concussion and DAI, which were 0.89, 0.55, and 0.25, respectively. The research was performed based on the animal experimental data simulated by the SIMon head model. It should be noted that the anatomical structure of the SIMon model was quite simplified compared to the real human head and the skull was assumed to be a rigid body. In this current study, the 50% risk of head AIS 4+ injury for MPS, CSDM_0.15_, and CSDM_0.25_ of 0.94, 0.6, and 0.285, respectively, in which these thresholds are very close to proposed by Takhounts et al. (2003, 2011, 2013).

According to the comparison with previous studies in the literature using different sources, it could be found that the threshold for head AIS 4+ injury in the elderly computed by the global kinematic criteria is generally lower than those of previous studies; for the brain tissue level criteria, the thresholds calculated were generally similar to those of previous studies except for the coup pressure. One reason is that all accidents involved elderly people, and another reason is that the injury threshold for all criteria in this study was obtained from the THUMS head model (Ver. 4.0.2); therefore, the model differences should be carefully considered in the future when applying the threshold.

### Limitation

The first limitation was that the accident cases are too limited and the number of head AIS 4+ cases (20 cases) and no head AIS 4+ cases (10 cases) were not equivalent in this research due to the high selection standards. The second limitation was that the kinematics could not be completely replicated according to the video information due to the limitations of the CPM model. Since not all deaths were analyzed anatomically, there existed some cases (six cases in total) without weight information. Admittedly, a more accurate numerical model also requires road user weight (Pascoletti et al., 2019b) in addition to the height and age, which is another limitation in this article. The variables in the regression models were only injury criteria and head AIS level, and did not include age and sex, mainly because of the small number of cases and the unbalanced proportion of sex and age groups (listed in Supplementary Table 1). Moreover, for some cases, the head collided with both the vehicle and the ground, but only the collision that caused the more severe head injury was included and the cumulative effect caused by another collision was not considered (Determine the collision that caused the more serious head injury using two aspects: Firstly, the specific position of the head impact with the vehicle and the ground can be derived from the video information. Then compared to the position of head injury in the injury report to determine whether the most serious head injury was caused by the vehicle or the ground. In addition, the values for each HIC were calculated for VRU during the vehicle impact and ground impact phases based on the THUMS 4.02 model, and the head injury values resulting from the vehicle and ground impact phases were compared to determine in which impact phase that caused the more severe head injury). And the THUMS head model represents a 50th male adult, the brain tissue mass and volume were not scaled according to the different ages and genders. The head injury models also did not consider the potential difference in tissue properties (e.g., skull stiffness), which was another limitation.

## Conclusion and Perspectives

Thirty in-depth VRUs accident cases with video records were reconstructed with high reliability by using a multi-body system (PC-Crash and MADYMO) and the THUMS (Ver. 4.0.2) head FE model. The kinematic-based injury criteria (linear acceleration, angular velocity, and acceleration, HIC) and brain tissue-based injury criteria (coup pressure, MPS, and CSDM) were investigated for predicting the head AIS 4+ injuries in elderly VRUs. The predictive ability of the logistic regression models was evaluated using the ROC curve and AUC, where the AUC ranged from 0.8 to 0.88, indicating a good correlation between all criteria and head AIS 4+ injury in the elderly. Thereby, the relevance of their capability to predict AIS 4+ brain injuries could therefore be compared with the AIS 4+ injury thresholds determined in the previous studies identified in the literature.

In this study, the determined injury threshold could alleviate the limited data on previously available brain tolerance. Also, the injury value acquired from in-depth real-world accident investigations could provide additional support for understanding brain injury mechanisms in elderly people. What’s more, the authors recommend that the comprehensive kinematic-based and tissue-based injury criteria should be considered for future VRUs’ safety studies.

## Data Availability Statement

The original contributions presented in the study are included in the article/Supplementary Material, further inquiries can be directed to the corresponding author.

## Author Contributions

YH conceptualization, methodology, writing original draft preparation, and writing reviewing and edition. HW software. HW, BW, and DP cases analysis, investigation, and joint writing the original draft preparation. YH and RT methodology, writing draft, and revising. KM conceptualization, supervision, and revising. All authors contributed to the article and approved the submitted version.

## Conflict of Interest

The authors declare that the research was conducted in the absence of any commercial or financial relationships that could be construed as a potential conflict of interest.
