# Supplementary material for: The Head AIS 4+ Injury Thresholds for the Elderly Vulnerable Road User Based on Detailed Accident Reconstructions
Source: Front Bioeng Biotechnol. 2021 Jun 23;9:682015. doi: 10.3389/fbioe.2021.682015 (PMC8261157; doi:10.3389/fbioe.2021.682015)
Supplement: Supplementary file 1 [file Data_Sheet_1.pdf]

Appendix

Table A1. Summary of the information for cases reconstructed. (ETW: electric two-wheeler; A: A-pillar, B: bonnet, W: windshield, G-ground)

Case ID.	Accident type	Impact velocity: Vehicle/VRU (km/h)	Vehicle information			VRU information			
			Vehicle type	Brand	Year built	Gender	Age	Height (cm)	Head impact area
C1	Pedestrian	36/8.57	Seden	Buick	2015	Male	74	165	B+G
C2	Pedestrian	30/5.04	Seden	BYD	2011	Male	69	168	A+G
C3	Pedestrian	28.6/2.16	MPV	Dongfeng	2008	Female	79	158	G
C4	Pedestrian	28.17/0	Sedan	Volkswegen	2011	Female	73	142	G
C5	Pedestrian	33.12/7.2	SUV	Zhongtai	2017	Female	80	154	G
C6	Pedestrian	40/0	Sedan	Volkswegen	2011	Male	80	166	B+G
C7	ETW	25.18/7.2	Sedan	Dongfeng	2008	Male	80	168	G
C8	ETW	61.1/19.7	MPV	Wuling	2015	Female	68	159	W+G
C9	ETW	72.02/17.02	Sedan	Audi	2014	Male	78	174	W+G
C10	ETW	45.4/19.6	Sedan	Chevrolet	2016	Female	66	160	A+G
C11	Bicycle	51.1/10.8	SUV	Beijing	2014	Male	67	170	B+G
C12	Bicycle	23.4/15	Sedan	Volkswegen	2008	Female	73	156	G
C13	ETW	34.2/18	Sedan	Roewe	2015	Female	60	160	W
C14	ETW	32.4/22.6	Sedan	Roewe	2011	Male	69	171	A+G
C15	Bicycle	36/7.56	Sedan	Volkswegen	2008	Male	90	164	W+G
C16	ETW	50.5/23.1	SUV	Audi Q5	2012	Male	64	164	G
C17	ETW	30.6/34.2	SUV	Nissan	2015	Male	72	165	G
C18	ETW	39.3/16.9	Sedan	Elantra	2016	Male	66	160	G
C19	ETW	25.2/3.6	Sedan	FIAT	2015	Male	70	170	B+G
C20	ETW	40/19.4	Sedan	Changan	2015	Male	80	160	G
C21	ETW	21.3/22	Sedan	Buick	2011	Female	64	154	G
C22	ETW	28/18	SUV	Citroen	2013	Male	64	162	G
C23	Pedestrian	45/10.1	Sedan	Volkswagen	2015	Male	75	160	A+G
C24	Bicycle	35.3/5.9	SUV	Porsche	2018	Female	71	160	B+G
C25	Pedestrian	41.3/3.88	MPV	Changan	2016	Male	66	165	G
C26	Pedestrian	45/3.4	SUV	Dongfeng	2014	Female	60	155	B+G
C27	Pedestrian	41.7/11.38	Sedan	Dongfeng	2019	Male	73	170	W+G
C28	ETW	36/25	Sedan	Mazda	2019	Male	62	166	W+G
C29	ETW	40.5/18	Sedan	Kia	2016	Male	74	166	B+G
C30	Pedestrian	53.1/0	Sedan	Chevrolet	2011	Male	79	165	W+G

Table A2. Summary of head injury records and AIS code. (Association for the Advancement Automotive Medicine, 2005)

Case ID	Head injury records	AIS code	MAIS
C1	Scalp hematoma	110402.1	1
C2	Cerebral hematoma, SDH, SAH, STH,DAI	140638.3, 140652.4, 140695.3, 110402.1, 140628.4	4
C3	SDH, SAH,SF,STH	140652.4, 140695.3, 150400.2, 110402.1	4
C4	SDH, SAH,CCL,ICH, Brain swelling	140654.4, 140695.3, 140611.3, 140656.5, 140660.3	5
C5	Concussion	161002.2	2
C6	DAI, CCL, Scalp abrasions	140628.4, 140611.3, 110202.1	4
C7	Brain contusion	140402.3	3
C8	SAH, Brain stem hemorrhage, Intraventricular hemorrhage	140695.3, 140210.5, 140678.2	5
C9	Brain contusion, SL	140616.4, 110606.3	4
C10	Brain contusion	140624.4	4
C11	Scalp abrasions, CH	110202.1, 140640.4	4
C12	SDH, SAH, SF, STH, BH	140652.4, 160695.3, 150400.2, 110402.1, 140202.5	5
C13	No injury	No injury	0
C14	SDH, SAH, SF, DAI, BH	140652.4, 140695.3, 150200.3, 140628.4, 140202.5	5
C15	SF	150202.3	3
C16	STH, SL	110402.1, 110602.1	1
C17	SDH, Concussion, STH, Cerebral infarction	140652.4, 161002.2, 110402.1, 140676.3	4
C18	SAH, SDH, SF, STH	140695.3, 140652.4, 150206.4, 110402.1	4
C19	No injury	No injury	0
C20	SAH, SDH, ICH, SF, Pneumocystis, BH	140695.3, 140656.5, 140649.4, 150400.2, 140682.3, 140202.5	5
C21	SL, SF, Facial bruises, CH	110606.3, 251000.1, 210202.1, 140645.4	4
C22	SDH, SAH, SF	140418.4, 140695.3, 150400.2	4
C23	SL, CCL, Scalp hematoma	110600.1, 140614.3, 110402.1	3

C24	Scalp hematoma	110402.1	1
C25	SDH, SAH, ICH, CCL, SF	140652.4, 140695.3, 140632.4, 140616.4, 150400.2	4
C26	SDH, SF, Traumatic brain herniation, SL	140656.5, 150402.2, 140202.5, 110600.1	5
C27	SF, SAH, SL, Cerebral infarction	150206.4, 140695.3, 110602.1, 140676.3	4
C28	SDH, SAH, SF, Pneumocystis	140654.4, 140695.3, 150000.2, 140682.3	4
C29	SDH, SAH, SL, SF, CCL, STH, Pneumocystis	140654.4, 140695.3, 110600.1, 150000.2, 140618.5, 110402.1, 140682.3	4
C30	Scalp hematoma	110402.1	1

**SDH: subdural hematoma, SAH: subarachnoid hematoma, STH: soft tissue hematoma, DAI: Diffuse axonal injury, SF: skull fracture, CCL: cerebral contusion/laceration, ICH: intracranial hematoma, SL: Scalp laceration, CH: Cerebral hematoma, BH: Brain herniation, SH: Traumatic brain herniation.*

Table A3. Summary of head velocities at the time before the head-to-vehicle and ground impact

Case ID	Time to Impact (ms)	Head linear velocity (m/s)			Head angular velocity (rad/s)		
		v_x	v_y	v_z	ω_x	ω_y	ω_z
C1	132	1.62	-3.92	-3.58	-35.48	-1.41	8.13
	1100	0.236	-4.533	-1.747	7.78	6.78	3.25
C2	162	-0.987	-6.516	-6.087	3.61	-19.15	-3.6
	403	-2.684	-6.35	-5.4	7.28	-3.65	-4.16
C3	683	0.548	-1.405	-4.26	0.013	1.014	-1.139
C4	400	1.326	-3.844	-5.517	-6.45	-1.326	-0.786
C5	499	0.757	-10	-7.107	-5.624	-0.096	0.92
C6	113	-1.85	0.73	-8.63	-61.76	-9.6	23.68
	1223	-0.305	-5.46	-3.15	14.46	-8.94	6.03
C7	804	0.86	-2.61	-7.07	10.9	0.76	1.61
C8	50	-4.1013	-14.02	-10.014	-93.76	-23.97	-9.652
	725	-3.117	-17.94	-4.71	0.024	-0.01	-3.25
C9	134	0.96	11.29	-8.73	-46.21	-3.52	0.44
	1139	0.74	-11.14	-5.36	-1.098	0.67	-2.35
C10	127	-1.82	7.46	-3.85	-30.89	0.16	-3.68
	1005	1.239	-5.772	-8.17	20.08	21.44	-4.85
C11	114	-0.791	-3.3494	-9.869	-64.716	-10.884	20.695
	993	-0.534	-7.339	-4.968	1.726	-1.326	-9.232
C12	826	2.845	3.169	-8.944	-22.25	8.703	-2.92
C13	301	-0.527	-4.262	-1.346	-6.605	0.65	9.404
C14	260	-0.854	1.3233	-1.346	-13.001	-4.327	5.829
	868	1.281	-5.891	-6.201	21.399	-16.6	6.943
C15	179	-0.28	6.7319	-4.2105	-19.79	-6.32	5.387
	1330	0.059	-4.764	-2.747	2.76	15.386	-3.691
C16	785	2.318	-2.279	-6.491	37.01	-9.789	5.175
C17	578	-6.159	8.219	-3.38	-3.61	-3.57	-9.69
C18	672	0.132	-2.971	-3.243	-7.87	0.84	18.25
C19	296	-0.32	0.517	-1.976	-6.921	-1.067	0.097
	1061	-0.11	-1.797	-1.866	-2.783	3.603	-0.37
C20	760	0.838	-2.017	-3.829	27.54	-0.412	0.54
C21	521	1.241	-6.518	-7.064	1.771	-1.92	-3.44
C22	867	0.6824	-5.6066	-5.0505	18.3916	-19.596	0.4512
C23	128	1.7666	-6.818	-7.0086	-25.59	-6.29	7.68
	654	3.7184	-5.8952	-2.7516	-2.95	-3.75	1.6
C24	235	0.0659	-0.7344	-4.7066	10.92	0.602	-0.05
	645	-1.1112	4.2457	-2.0985	16.94	-4.59	0.19
C25	1070	8.0777	-2.1697	-8.121	-11.93	58.76	-19.34
C26	76	1.6215	3.4809	-7.4496	-54.036	-1.275	-8.004
	1026	0.661	-7.1075	-5.9919	51.81	-8.114	5.484
C27	152	0.9808	6.8129	-2.2648	-21.29	-20.35	-1.97
	684	1.676	-2.073	-7.205	6.661	0.847	2.766
C28	300	0.9221	-2.6294	-0.0923	-10.37	5.2	-4.96
	862	3.0155	-6.5896	-5.3771	-0.57	1.91	4.19
C29	206	-2.405	0.424	-4.077	-12.055	7.993	6.319
	1706	-0.3588	-3.3464	-2.1135	-1.6328	11.114	-9.4361
C30	95	-1.0795	7.2519	-9.0019	-70.75	-9.03	2.02
	1269	-1.3353	-11.474	-3.2994	-7.54	2.08	1.35

Table A4. Summary of calculated head injury parameters for cases reconstructed

Case ID		*Head kinematic-based criteria*				*Tissue level injury criteria*			
		Ang_vel (rad/s)	*Ang_acc (rad/s2)*	*Lin_acc (g)*	*HIC_{15}*	*Pressure (kPa)*	*MPS*	*CSDM (0.15)*	*CSDM (0.25)*
C1		20.5	6080	137	321	344	0.65	0.18	0.009
C2		68.3	31382	411	2162	1198	1.64	0.953	0.659
C3		63.1	12693	216	1423	1313	2	0.975	0.847
C4		85.7	14052	394	2998	1480	2.46	0.96	0.614
C5		25	20465	166	1522	397	0.84	0.586	0.358
C6		68.03	26527.7	176.8	1170.4	479.4	0.755	0.412	0.0972
C7		20.4	11702	370	1808	2402	1.22	0.633	0.243
C8		97.262	20152	319.58	3311	3032.67	2.28	0.996	0.975
C9		70.1	24614	279	1841	2638	1.387	0.831	0.699
C10		51	31859	530	2897	3618	0.77	0.888	0.494
C11		21.3	22900	319	2630.3	195.1	0.822	0.5298	0.1813
C12		48.9	36688	513	2889	3587	0.729	0.855	0.4673
C13		25	5550	160	214	319	0.77	0.561	0.189
C14		52.6	33469	372	2399	1467	2.165	0.9367	0.5924
C15		43.5	17093	230	817	637	0.8	0.619	0.231
C16		48.7	29500	388	4238	1123	2.02	0.906	0.603
C17		66.2	21629	213	1153	971	2.248	0.9607	0.7552
C18		38.614	14428	254.69	1440	408.7	1.047	0.802	0.412
C19		17.12	6730.8	157.89	377.5	177.71	0.966	0.621	0.282
C20		19.1	24900	530.33	2389	1128.48	1.822	0.7392	0.3917
C21		35.84	25817	290.8	1217	803.8	1.884	0.714	0.376
C22		43.395	23263	330.32	3031	2160	0.9642	0.8421	0.4247
C23		35.24	17137.9	298.67	1476.2	815.1	1.228	0.9186	0.3018
C24		34.73	10615.1	154.89	759.9	78.44	0.322	0.037	0.00097
C25		32.527	14802	246	1200	372.9	1.71	0.683	0.304
C26		65.21	19676	175.38	1000.4	271.8	0.956	0.5372	0.245
C27		27.54	14337	358.23	2916	936.7	1.899	0.9739	0.8128
C28		37.52	25603.2	176.08	1103.8	612.3	1.91	0.9419	0.7524
C29		15.16	12802.1	163.75	486.7	591.9	1.47	0.8562	0.49085
C30		14.4	9770	73	103	621.6	1.016	0.6101	0.3604
No head AIS 4+	Min	14.4	5550	73	103	78.4	0.32	0.04	0.001
	Ave	28.7	12267	187.8	823.1	417.1	0.93	0.56	0.245
	Max	43.5	20465	298.7	1522	815.1	1.71	0.92	0.41
Head AIS 4+	Min	15.2	11702	163.8	486.7	195.1	0.73	0.41	0.097
	Ave	50.3	23178	326.3	2153.2	1500.5	1.57	0.82	0.536
	Max	97.3	36688	530.3	4238	3618	2.46	0.996	0.975
*Magnitude of the difference		21.6	10911	138.5	1330.1	1083.4	0.64	0.26	0.291

* Magnitude of the difference=Average $_{(AIS \geq 4)}$-Average $_{(AIS<4)}$

Figure A1: Comparison of reconstructed kinematics with video screenshots.

Case ID: C1

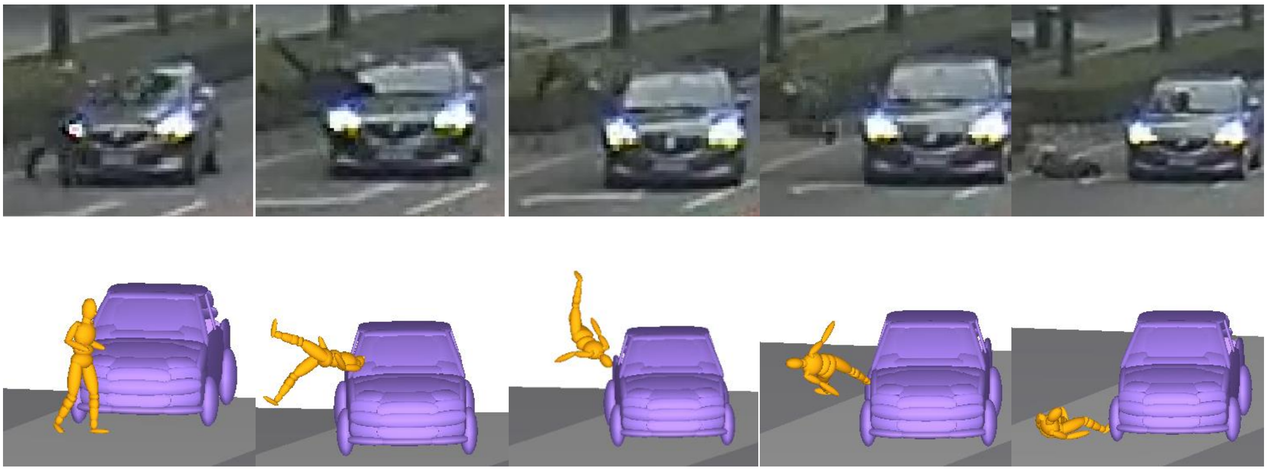

0 ms **132 ms** **520 ms** **664 ms** **924 ms**

Case ID: C2

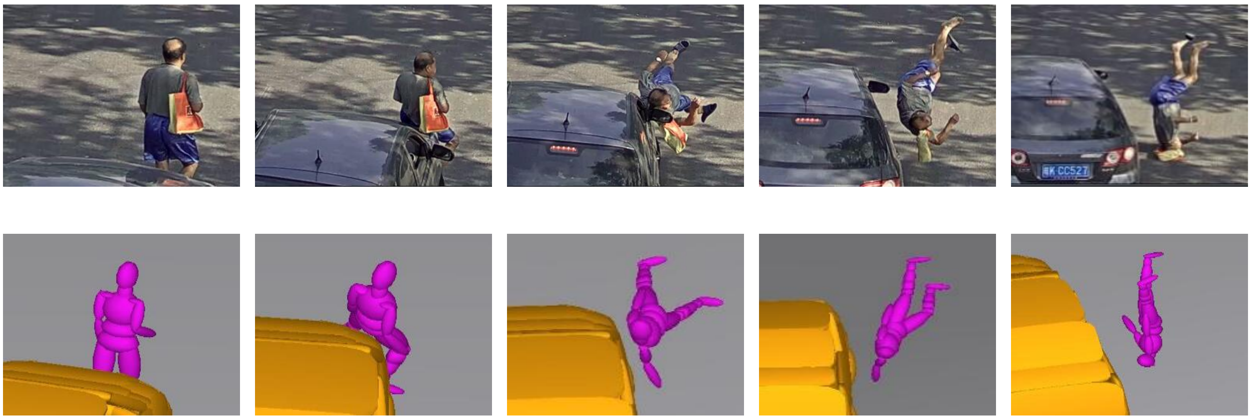

0 ms **160 ms** **280 ms** **360 ms** **480 ms**

Case ID: C3

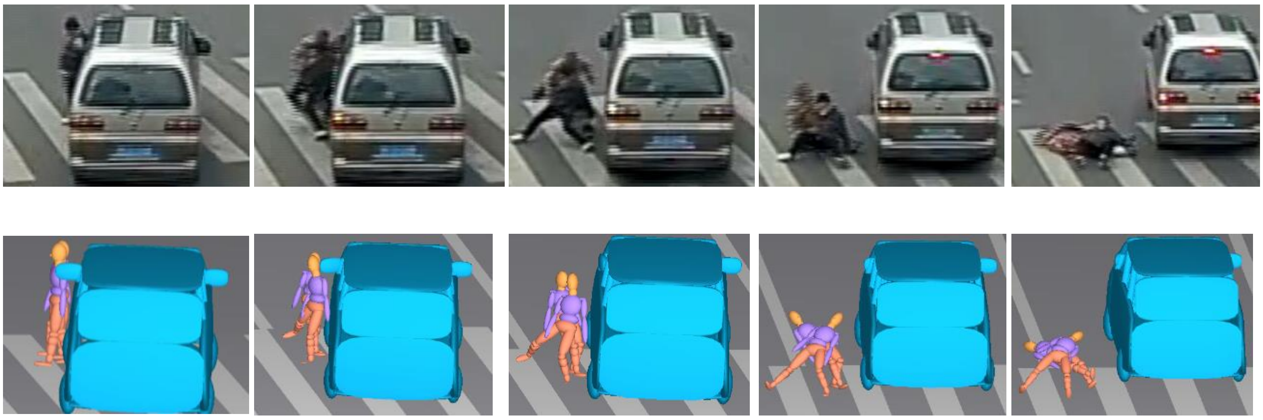

0 ms **120 ms** **280 ms** **480 ms** **600 ms**

Case ID: C4

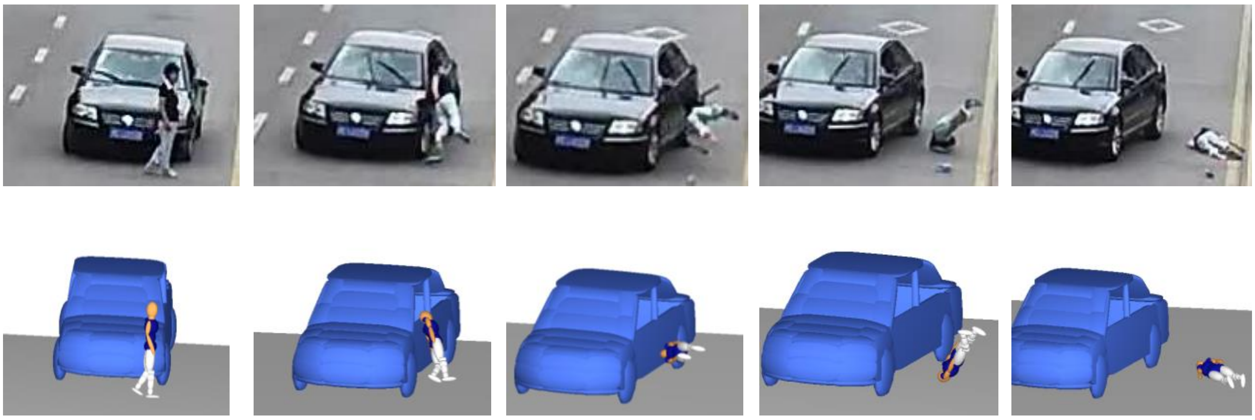

0 ms **180 ms** **280 ms** **398 ms** **780 ms**

Case ID: C5

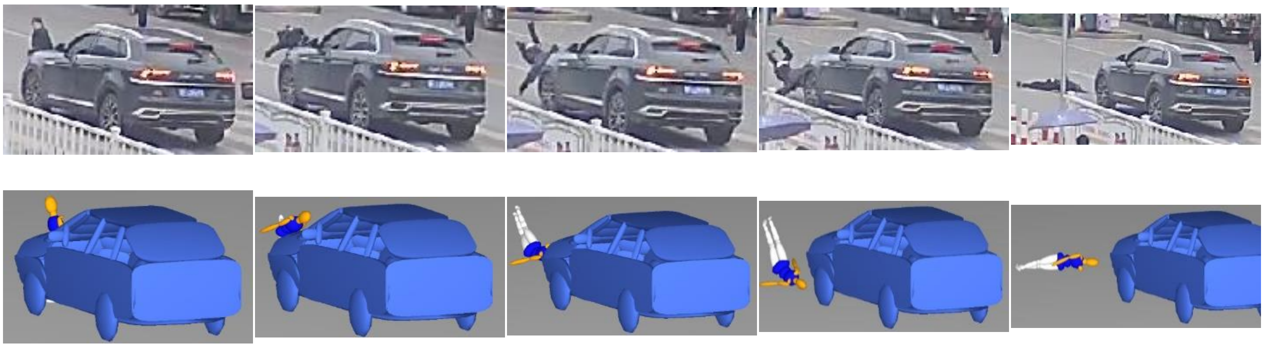

0 ms **208 ms** **417 ms** **489 ms** **2000 ms**

Case ID: C6

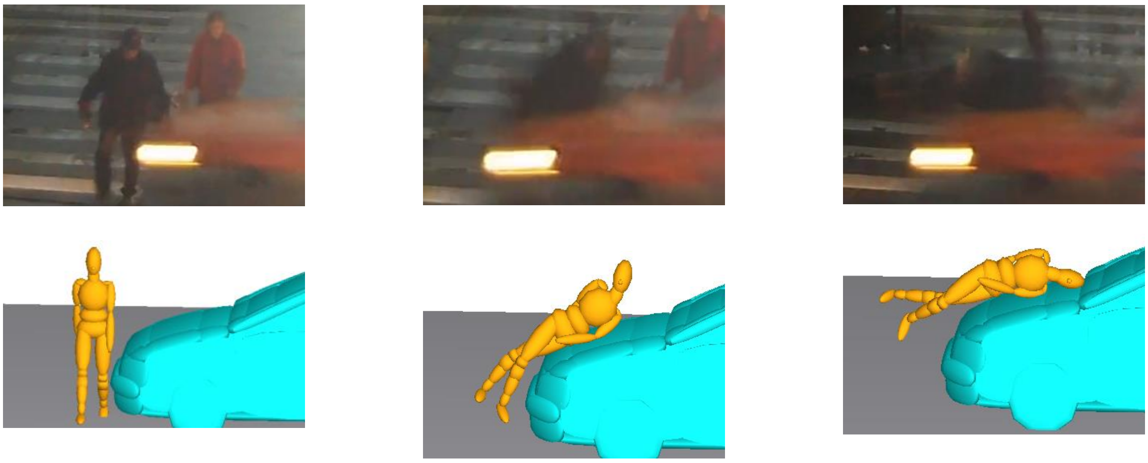

0 ms **80 ms** **114 ms**

Case ID: C7

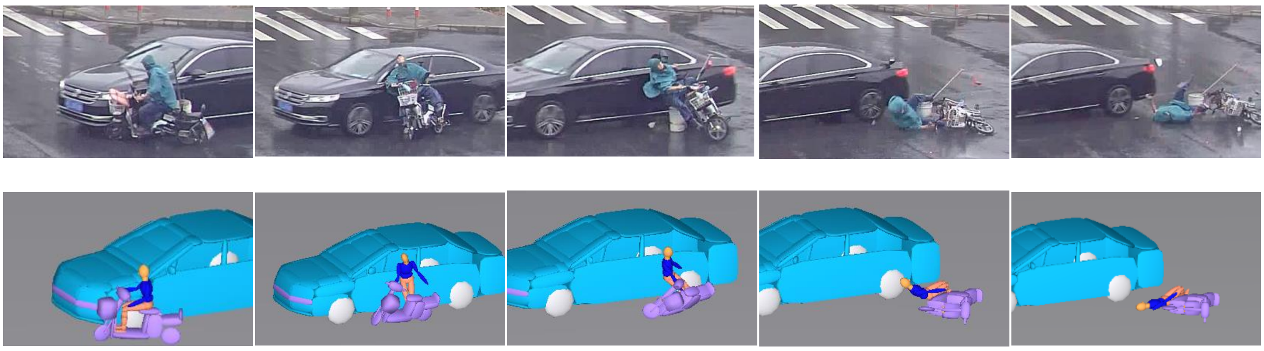

0 ms **200 ms** **360 ms** **520 ms** **600 ms**

Case ID: C8

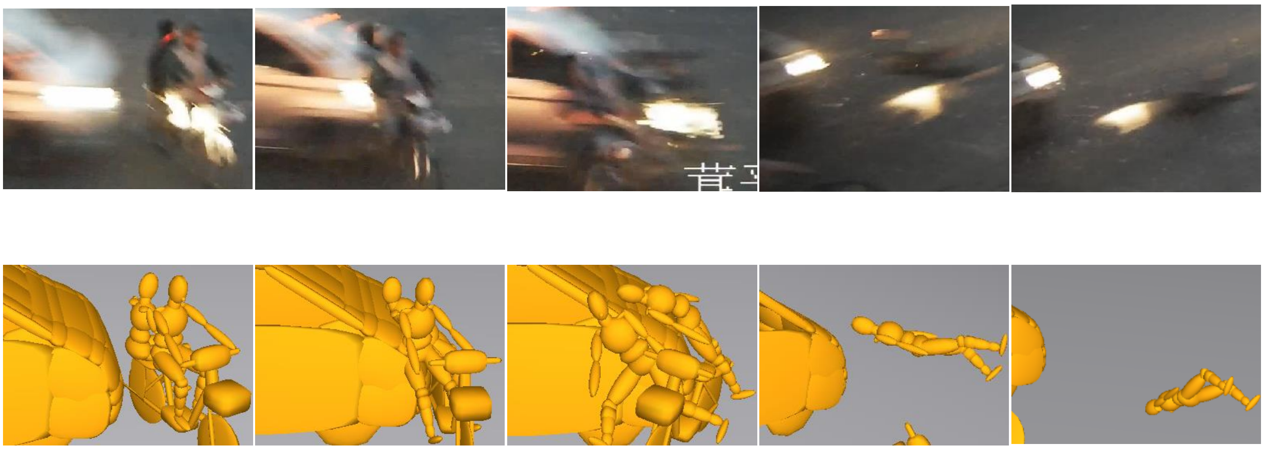

0 ms **50 ms** **100 ms** **325 ms** **400 ms**

Case ID: C9

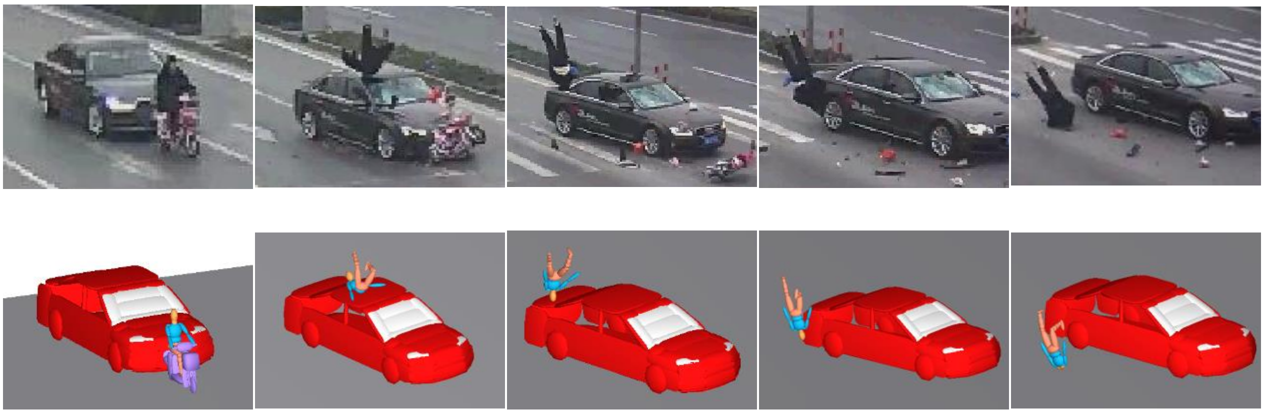

0 ms **400 ms** **800 ms** **960 ms** **1100 ms**

Case ID: C10

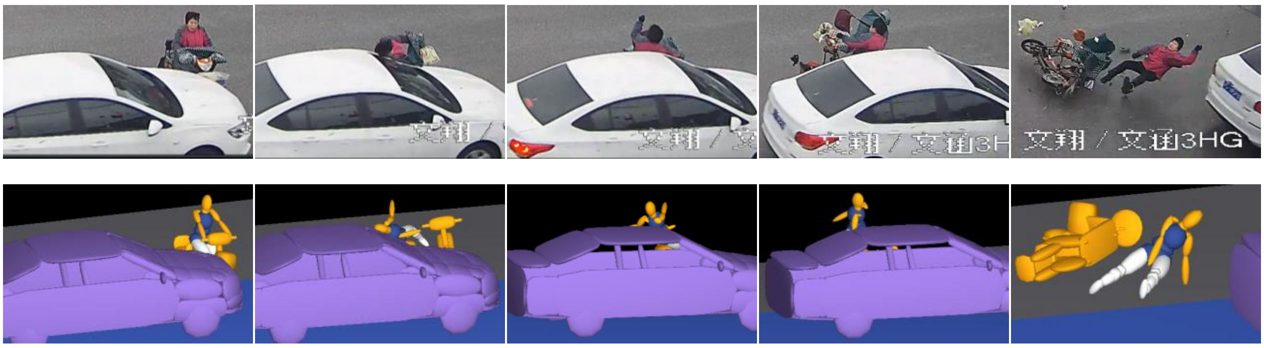

0 ms **126 ms** **258 ms** **525 ms** **850 ms**

Case ID: C11

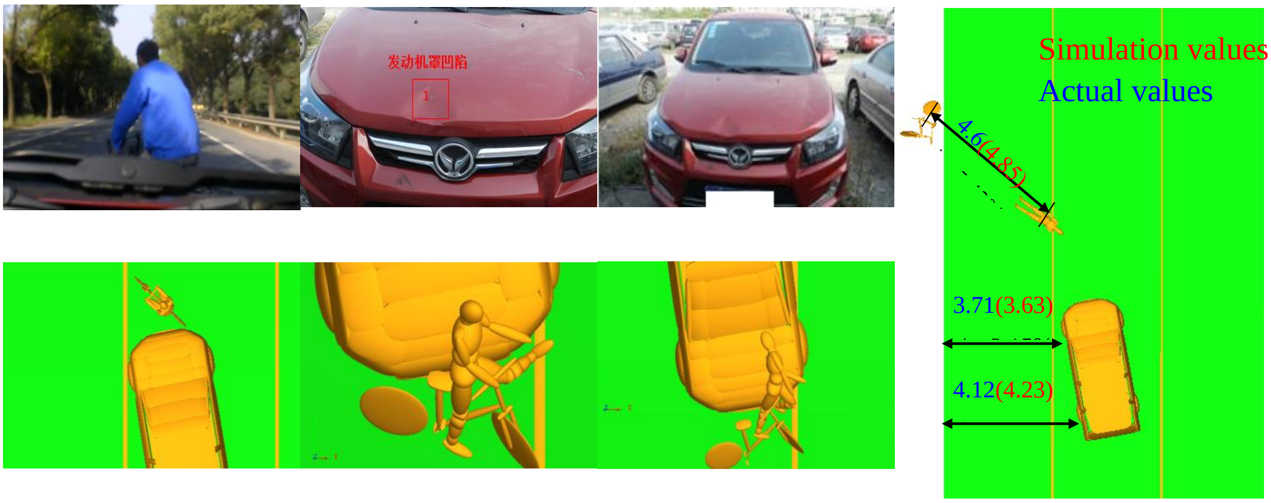

0 ms **32 ms** **164 ms** **Final position**

Case ID: C12

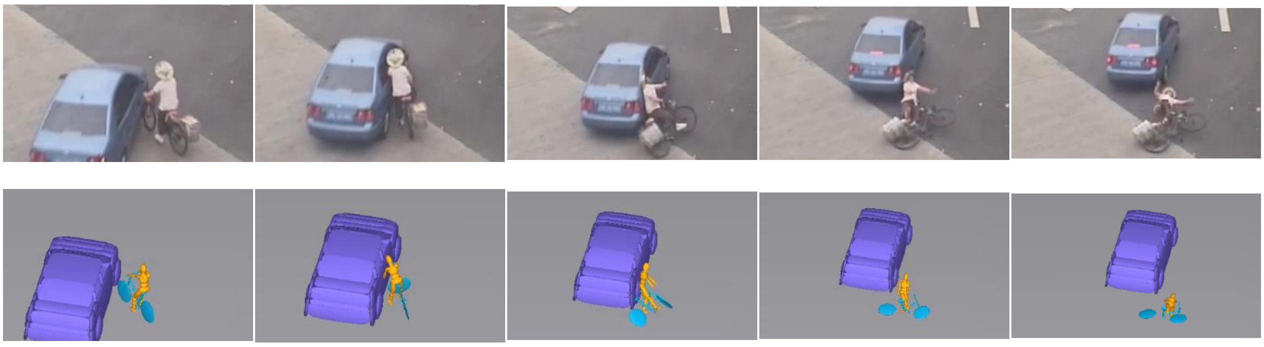

0 ms **240 ms** **480 ms** **720 ms** **840 ms**

Case ID: C13

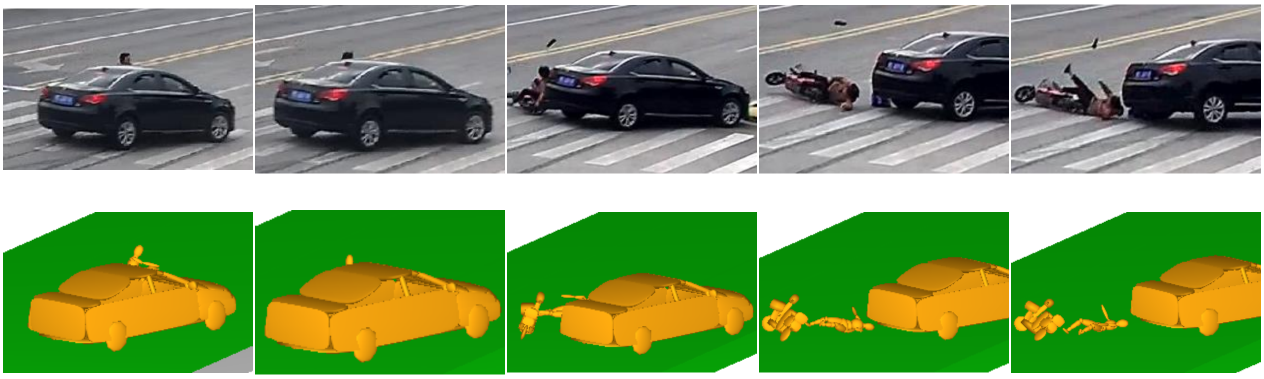

0 ms 80 ms 320 ms 480 ms 560 ms

Case ID: C14

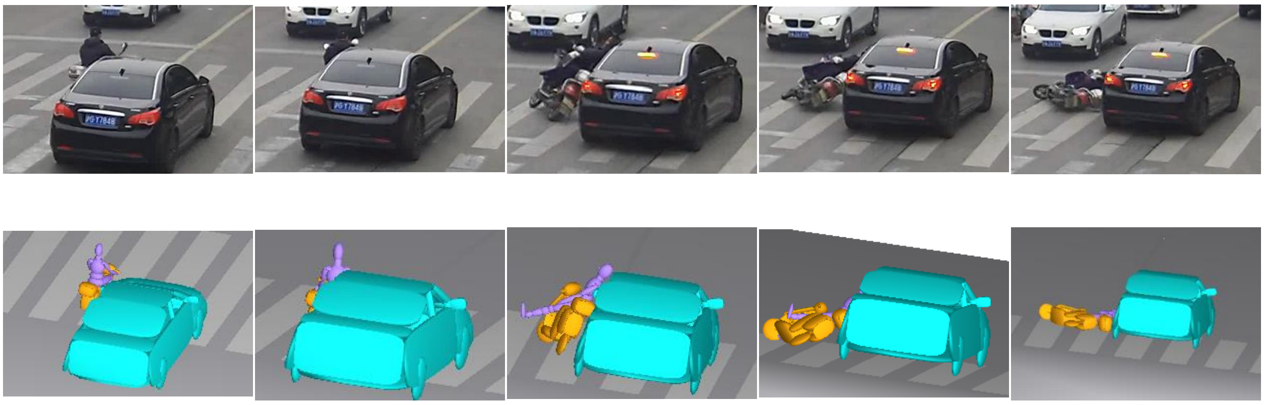

0 ms 75 ms 240 ms 840 ms 982 ms

Case ID: C15

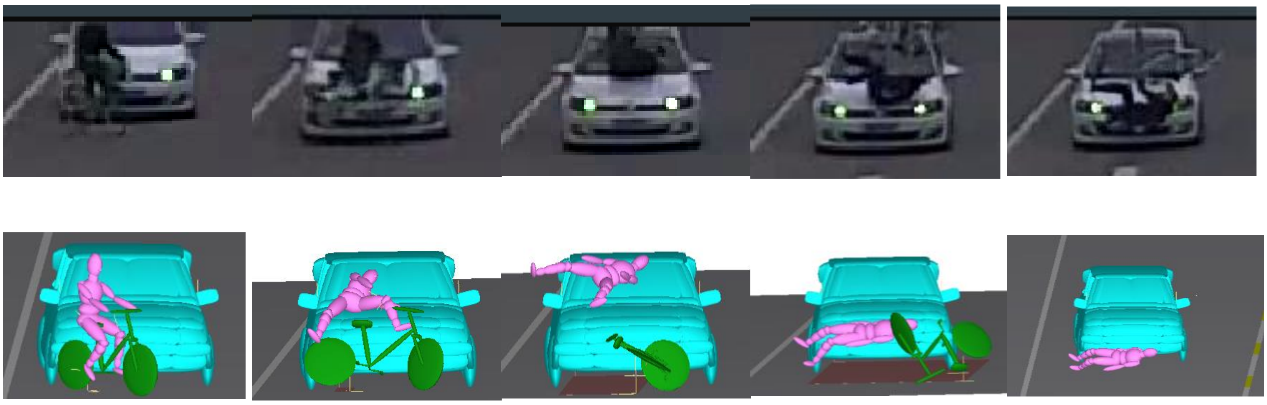

0 ms 185 ms 250 ms 500 ms 650 ms

Case ID: C16

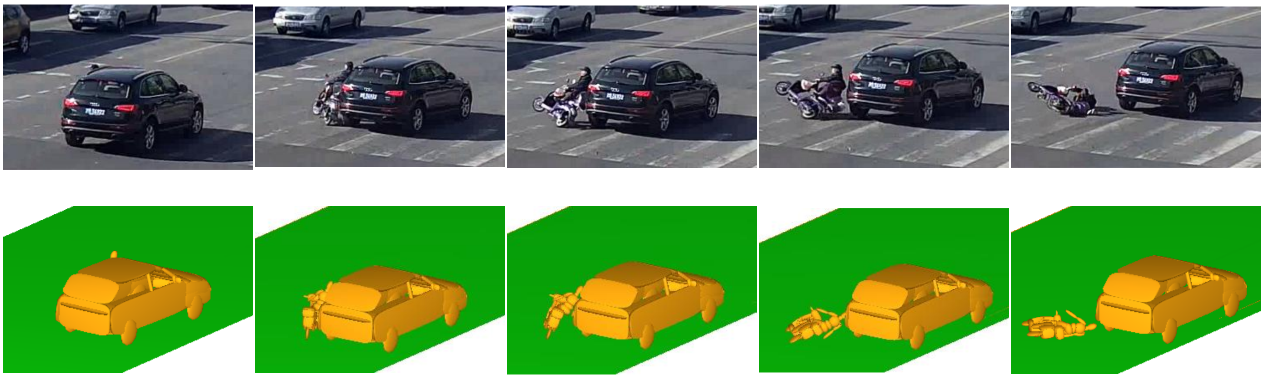

0 ms 200 ms 490 ms 530 ms 740 ms

Case ID: C17

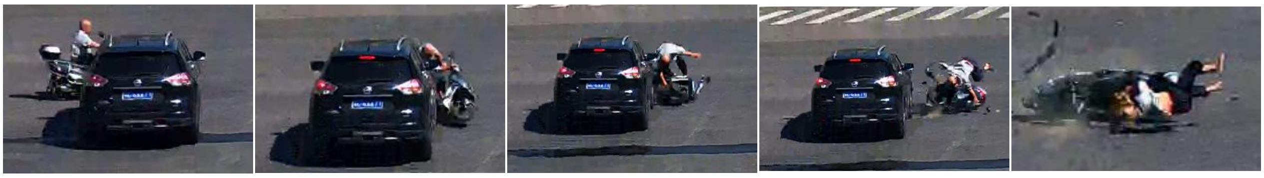

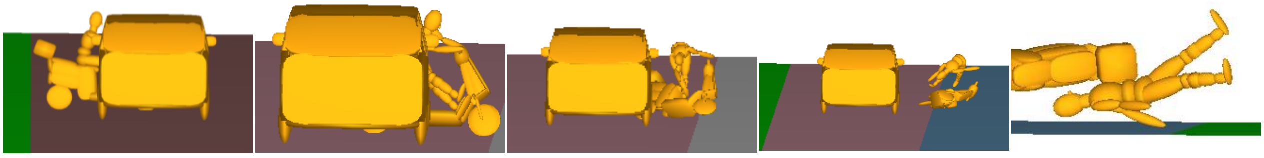

0 ms 50 ms 100 ms 200 ms 575 ms

Case ID: C18

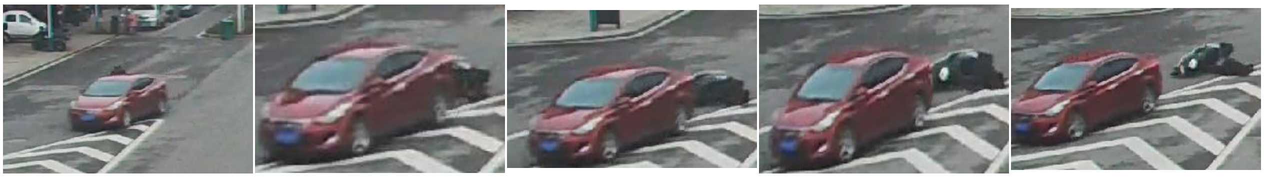

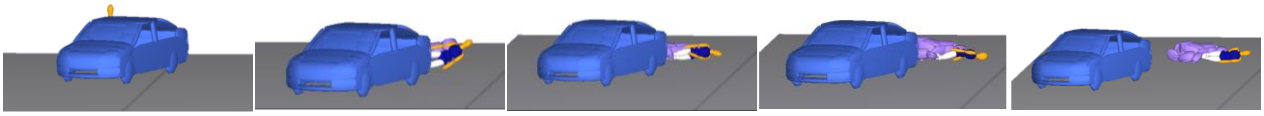

0 ms 120 ms 160 ms 240 ms 360 ms

Case ID: C19

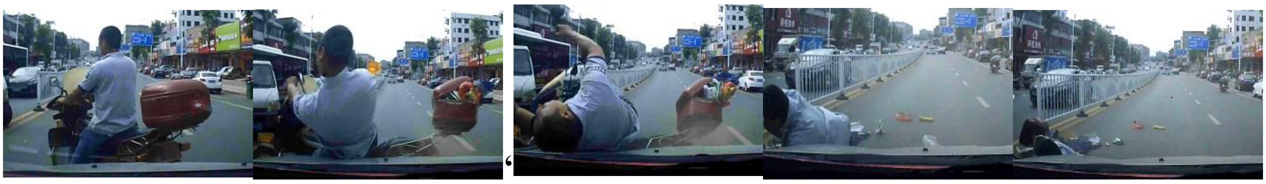

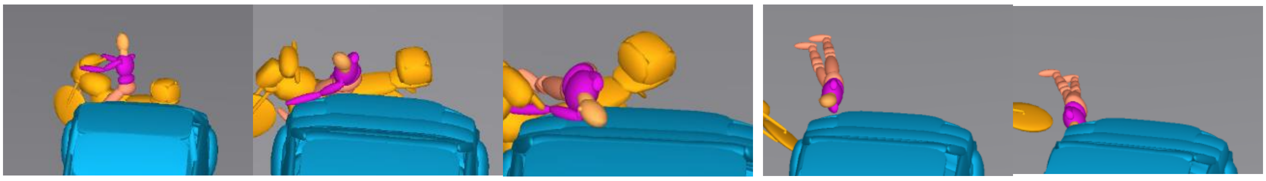

0 ms 193 ms 298 ms 696 ms 844 ms

Case ID: C20

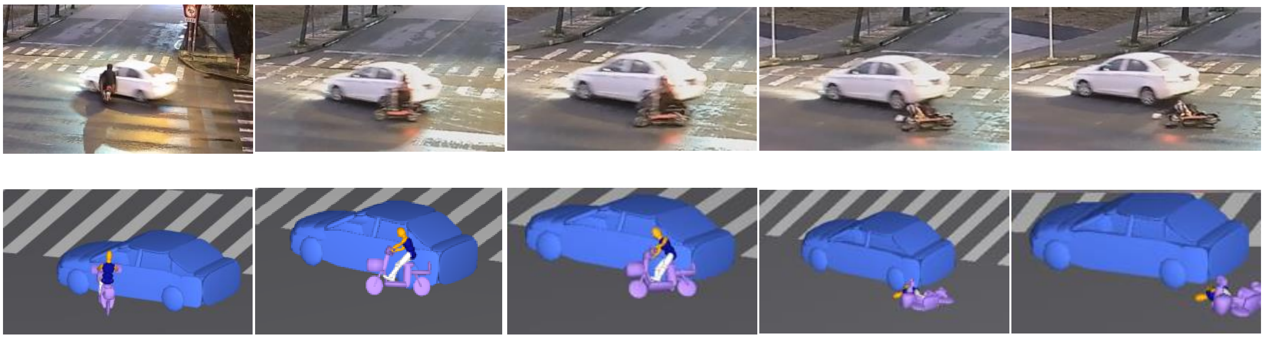

0 ms 310 ms 430 ms 830 ms 1050 ms

Case ID: C21

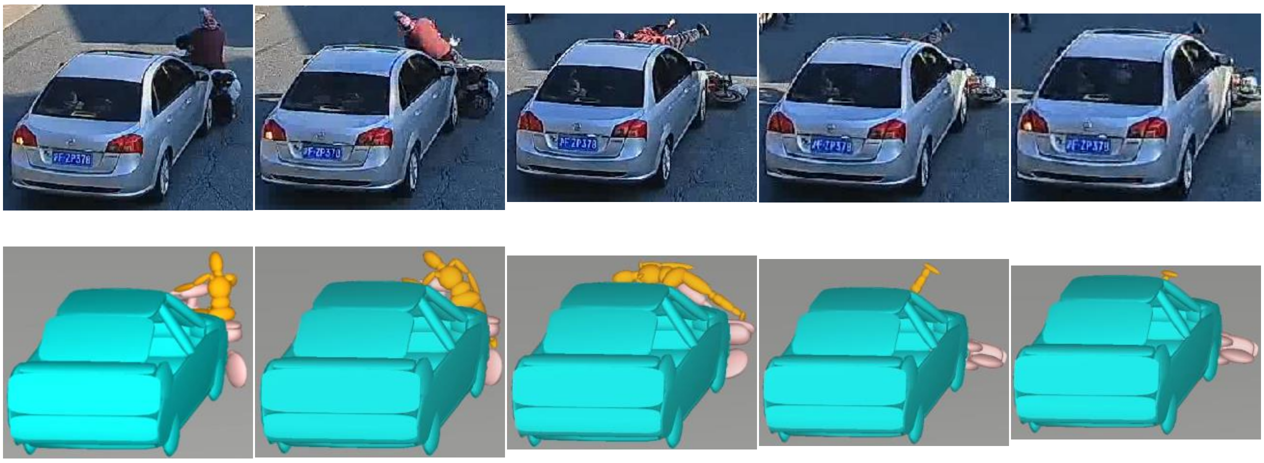

0 ms 70 ms 238 ms 466 ms 550 ms

Case ID: C22

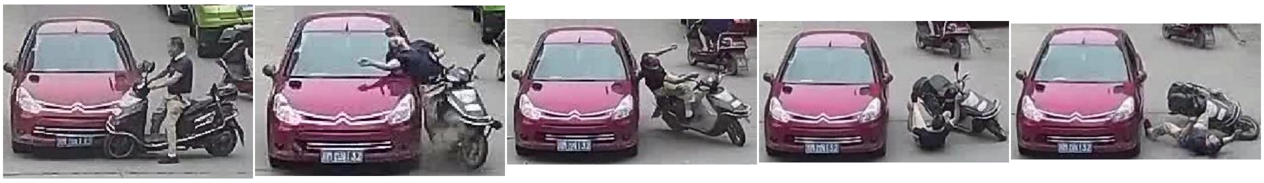

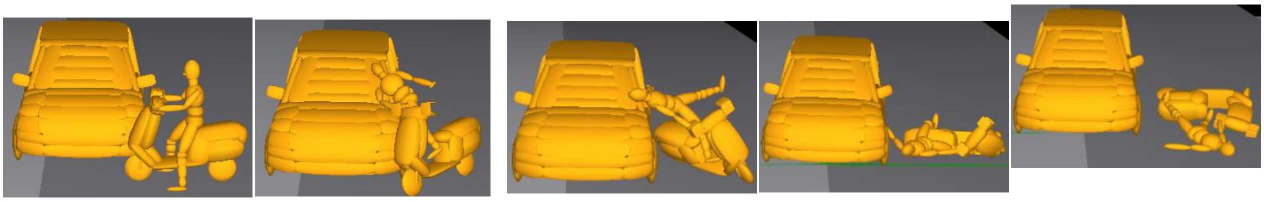

0 ms | 185 ms | 407 ms | 666 ms | 814 ms

Case ID: C23

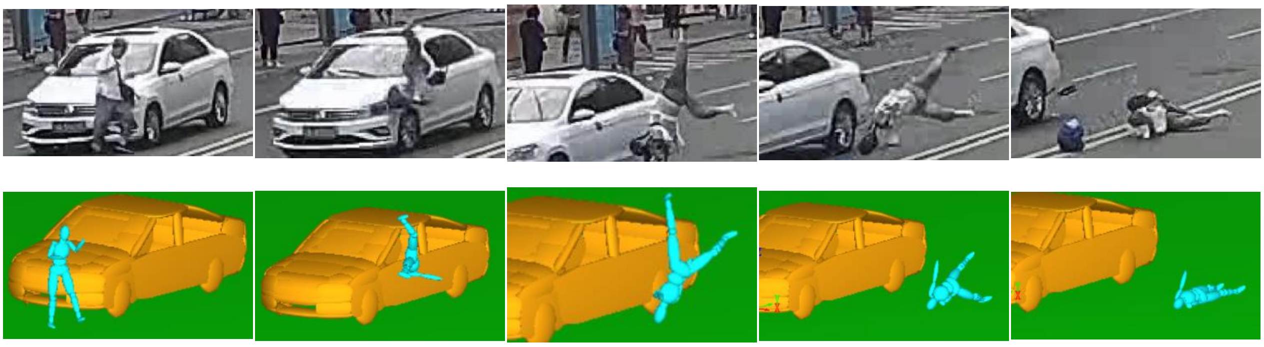

0 ms | 240 ms | 380 ms | 540 ms | 640 ms

Case ID: C24

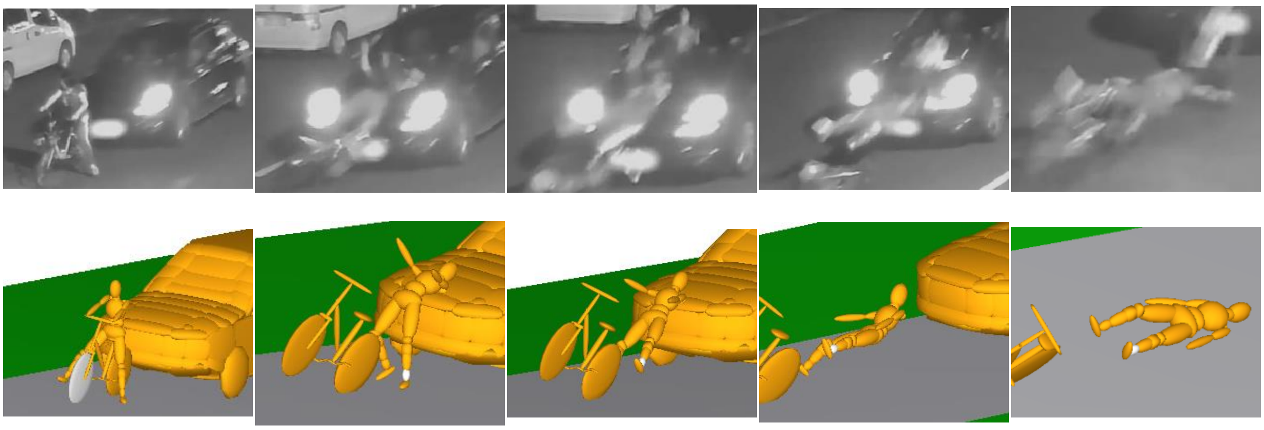

0 ms | 285 ms | 375 ms | 495 ms | 640 ms

Case ID: C25

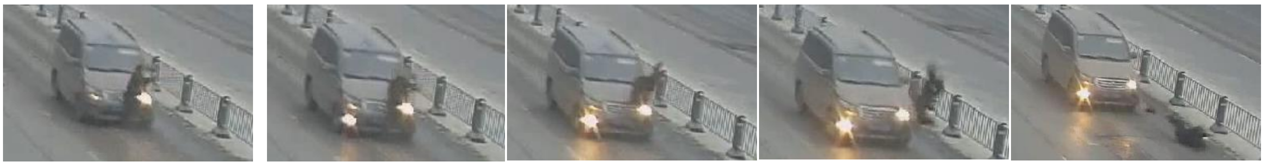

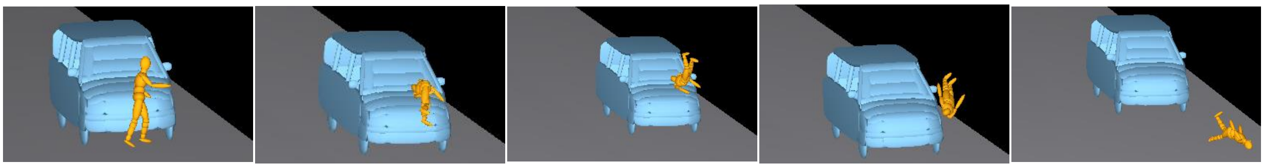

0 ms **160 ms** **280 ms** **640 ms** **1080 ms**

Case ID: C26

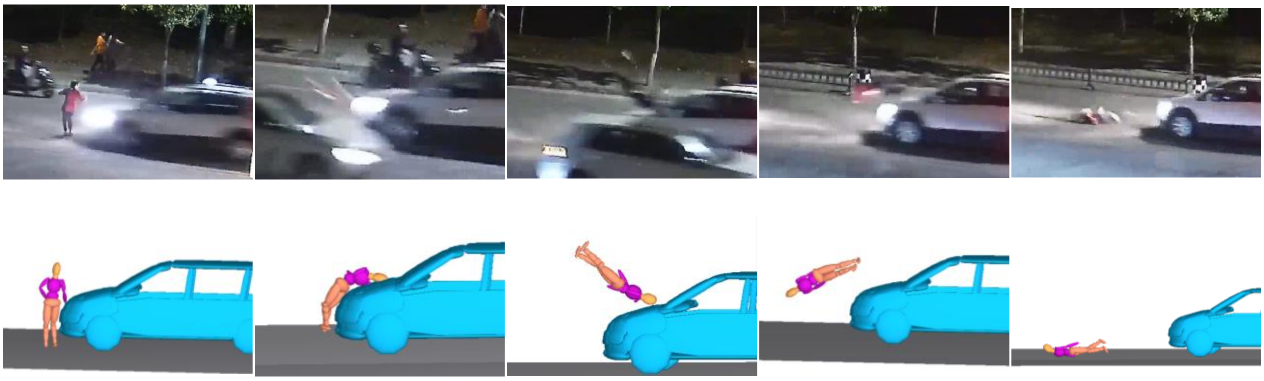

0 ms **72 ms** **276 ms** **672 ms** **1060 ms**

Case ID: C27

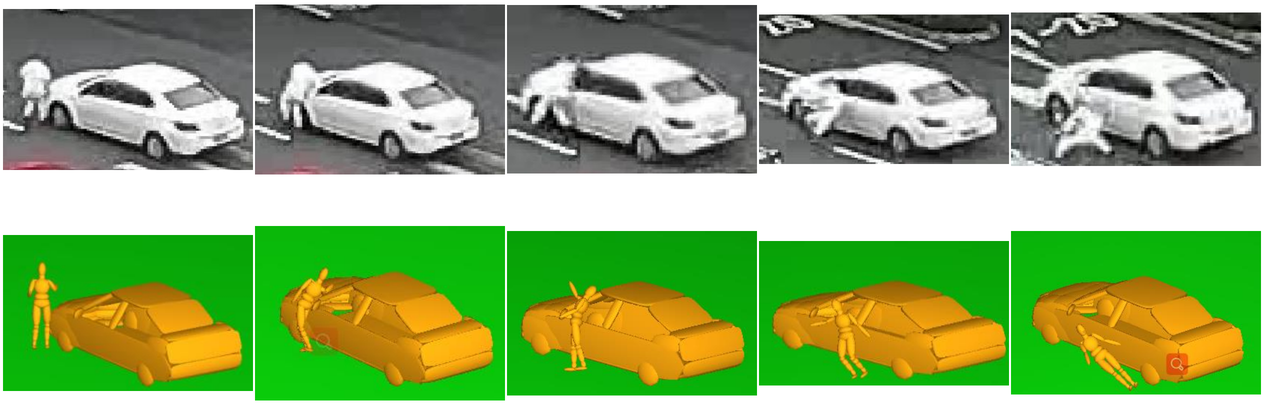

0 ms **115 ms** **220 ms** **390 ms** **530 ms**

Case ID: C28

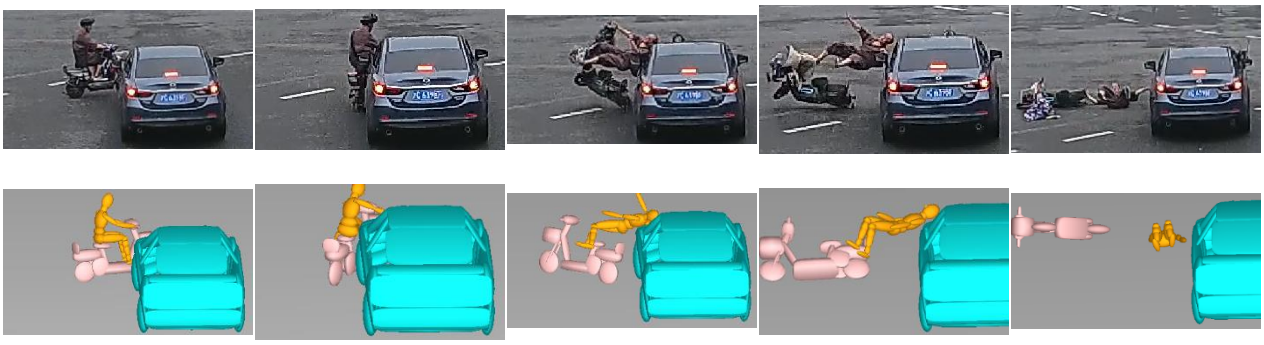

0 ms **114 ms** **412 ms** **630 ms** **858 ms**

Case ID: C29

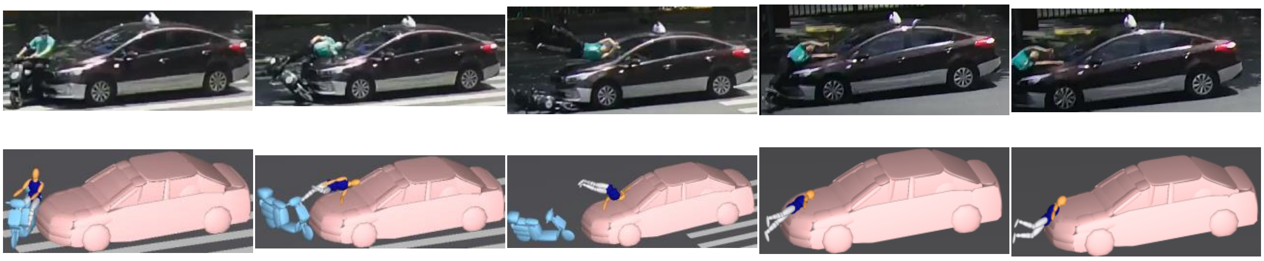

0 ms **173 ms** **310 ms** **820 ms** **1065 ms**

Case ID: C30

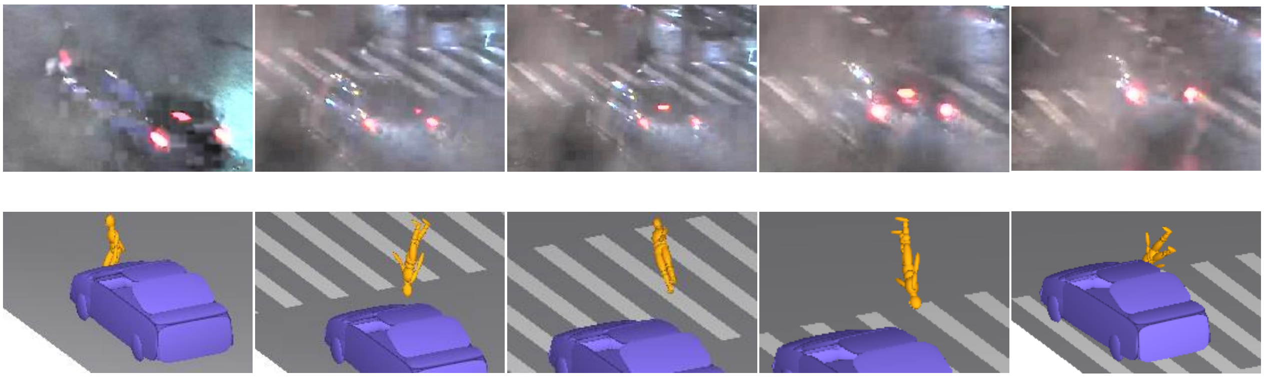

0 ms **302 ms** **704 ms** **911 ms** **1239 ms**
